# Low Dose Radiation Therapy, Particularly with 0.5 Gy, Improves Pain in Degenerative Joint Disease of the Fingers: Results of a Retrospective Analysis

**DOI:** 10.3390/ijms21165854

**Published:** 2020-08-14

**Authors:** Anna-Jasmina Donaubauer, Jian-Guo Zhou, Oliver J. Ott, Florian Putz, Rainer Fietkau, Ludwig Keilholz, Udo S. Gaipl, Benjamin Frey, Thomas Weissmann

**Affiliations:** 1 Department of Radiation Oncology, Universitätsklinikum Erlangen, Friedrich-Alexander-Universität Erlangen-Nürnberg (FAU), 91054 Erlangen, Germany; Anna-Jasmina.Donaubauer@uk-erlangen.de (A.-J.D.); jianguo.zhou@fau.de (J.-G.Z.); Oliver.Ott@uk-erlangen.de (O.J.O.); Florian.Putz@uk-erlangen.de (F.P.); Rainer.Fietkau@uk-erlangen.de (R.F.); Udo.Gaipl@uk-erlangen.de (U.S.G.); Benjamin.Frey@uk-erlangen.de (B.F.); 2 Department of Oncology, The Second Affiliated Hospital of Zunyi Medical University, Zunyi 563000, China; 3 Department of Radiotherapy, Clinical Center Bayreuth, 95447 Bayreuth, Germany; ludwig.keilholz@klinikum-bayreuth.de

**Keywords:** low-dose radiation therapy, LDRT, degenerative joint disease of the fingers, osteoarthritis, chronic degenerative and inflammatory diseases, subjective pain level

## Abstract

Low-dose radiation therapy (LDRT) has been successfully established for decades as an alternative analgesic treatment option for patients suffering from chronic degenerative and inflammatory diseases. In this study, 483 patients were undergoing LDRT for degenerative joint disease of the fingers and thumb at the University Hospital Erlangen between 2004 and 2019. Radiotherapy was applied according to the German guidelines for LDRT. Several impact factors on therapeutic success, such as the age and gender, the number of affected fingers, the single and cumulative dose, as well as the number of series, were investigated. In summary, 70% of the patients showed an improvement of their pain following LDRT. No significant impact was found for the factors age, gender, the number of series or the cumulative dosage. Patients with an involvement of the thumb showed a significantly worse outcome compared to patients with an isolated affection of the fingers. In this cohort, patients receiving a single dose of 0.5 Gy reported a significantly better outcome than patients receiving 1.0 Gy, strongly suggesting a reduction in the total dose. In summary, LDRT is a good alternative treatment option for patients suffering from degenerative and inflammatory joint disease of the fingers.

## 1. Introduction

As the median age of the population in the western world is constantly rising, arthritis as the leading degenerative joint disease is showing increasing prevalence. Amongst the different forms of arthritis, osteoarthristis is the most common form, leading to destructive processes in numerous tissues of the affected joints during progression [1]. Osteoarthritis can affect multiple joints, but osteoarthritis of the hand is amongst the most common forms with a high clinical burden. As the hand consists of numerous joints that can be affected, hand osteoarthritis is a complex and heterogeneous disorder. While not being attributed to a specific cause, the development of osteoarthritis and degenerative joint disease of the fingers in general, seems to be supported by multiple risk factors. Amongst these risk factors, a high age, female gender, obesity and mechanical forces are the most prominent ones. Generally, the disease shows a slow but steady progression. During the course of the disease the whole joint becomes involved, leading to the degradation of cartilage and bone, as well as to synovial inflammation. In summary, osteoarthritis and degenerative joint disease of the fingers are causing pain as well as a loss of strength, flexibility and function in the affected joint [2,3].

The course of the disease shows a steady progression, often affecting several joints, following a characteristic pattern. Finger osteoarthritis can show a symmetric clustering, affecting both hands in the same manner. Additionally, rather than affecting all fingers, several joints of one digit show symptoms first. The most frequently affected joints include the joints of the thumb, the first interphalangeal joint, the first metacarpophalangeal joint and the first carpometacarpal joint. In advanced disease stages, the osteoarthritis of several fingers leads to an impaired function of the whole hand, resulting in a diminished quality of life [2,4].

Therapeutic options for osteoarthritis and degenerative joint diseases of the fingers range from conservative to operative approaches, depending on disease stage and the subjective burden of the patient, primarily aiming for pain reduction as well as a deceleration of disease progression. There is a broad range of medical treatment options, ranging from non-pharmacological treatments, such as physical therapy or orthoses to non-steroidal anti-inflammatory drugs (NSAIDs) and intraarticular injections [1,2,3,5]. Nonetheless, many of these drugs have serious side effects. Even though patients can benefit from numerous therapeutic options, about 25% of all patients do not respond to these therapies or lose their responsiveness over time, as the disease shows a steady progression [6]. This is why alternative treatment options with low side effects are urgently needed for the treatment of chronic degenerative joint diseases of the fingers, such as osteoarthritis.

In Germany, the use of low-dose radiation therapy (LDRT) for enthesiopathies and chronic degenerative and inflammatory diseases, such as painful arthritis and arthrosis has successfully been established for decades and is gaining acceptance by orthopedic specialists due to the satisfying results achieved [7]. Especially patients that are refractory for the classical treatment options may benefit from LDRT, as the therapy induces long-lasting pain-relieving effects. During therapy, patients are irradiated with fractionated low doses of X-rays with a single dose between 0.5 Gy and 1.0 Gy [7,8]. This serial radiation leads to a pain reduction in the affected joint and ameliorates inflammatory processes [9,10,11,12]. Those effects last several months, confirming the therapeutic benefits of LDRT in the therapy of chronic degenerative and inflammatory diseases [13,14,15]. Even though, LDRT offers promising analgesic effects, many health professionals do have concerns regarding radiation-induced side effects, such as the induction of cancer. By now, no increase in the cancer rate after LDRT has been reported so far [16,17]. In the case of the radiation therapy of the upper extremities, the radiation exposure to the gonads also plays a minor role, as the dose is in the same range as during a standard X-ray examination. Especially in older patients that are no longer planning on having children, gonad exposure only plays a minor role [18]. Therefore, LDRT is a favorable alternative treatment option. Especially for patients in a burnt-out phase that did not respond to the standard treatment options, LDRT might induce good pain-relieving effects.

Despite the fact that LDRT is successfully applied in the clinic, the underlying molecular and immunological effects remain mostly elusive and are still subject to current research. Many cell types and their osteoimmunological mechanisms are regulated by LDRT, such as the bone resorbing activity of osteoclasts, the inflammatory phenotype of macrophages or the adhesion of infiltrating immune cells to endothelial cells. At least in part, these mechanisms may be responsible for the pain-reliving and anti-inflammatory effects of LDRT. Over the years, numerous preclinical and clinical studies provided evidence for those pain-relieving and molecular effects [17,19,20]. Despite a vast amount of positive data on LDRT for arthritis, osteoarthritis and other chronic degenerative and inflammatory diseases in different locations are available, recent published data of first placebo-controlled trials raised doubts towards an anti-inflammatory or pain relieving effect of LDRT [21,22].

In contrast, the presented study further supports the therapeutic benefits of LDRT for the treatment of chronic degenerative joint diseases of the fingers. Between 2004 and 2019, nearly 500 patients underwent LDRT for the treatment of chronic degenerative joint disease/osteoarthritis of the fingers at the Department of Radiation Oncology at the University Hospital Erlangen. Our detailed retrospective analyses have indicated that LDRT, particularly with a single dose per fraction of 0.5 Gy, improves pain levels in irradiated fingers, but not in the thumb.

## 2. Results

**Figure 1:**
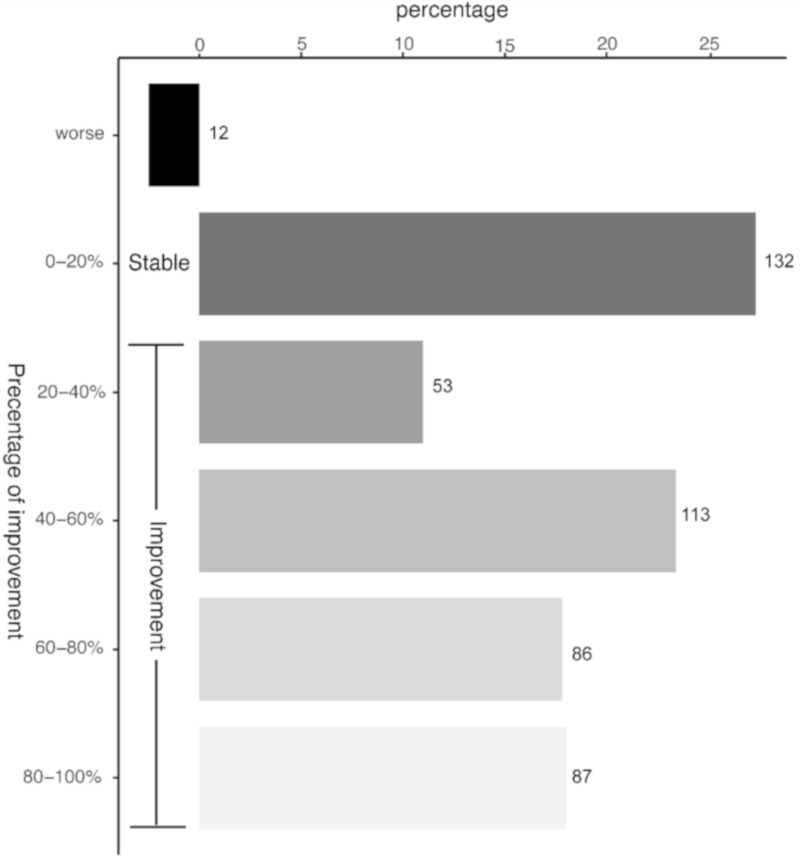
Pain improvement following LDRT. Patients scored the subjective improvement of their pain level in percentage of improvement in regard of their initial pain level before therapy. The pain levels were determined after the last LDRT session, as well as 12 and 24 weeks after LDRT, if available. The figure depicts the best therapeutic response of the patients independent of the time point and the single and cumulative dose. Patients with an improvement up to 20% were considered as those with a stable disease. The numbers at the bars depict the number of patients of the analysis with the corresponding percentage of improvement.

The patients scored their subjective pain reduction directly after the last radiotherapy session and, if available, after three and six months. In order to depict the therapeutic success, the best therapeutic response independent of the time point of data collection and the cumulative dose is presented in Figure 1.

The majority of the patients (about 70%) reported a subjective improvement of their symptoms of more than 20%. This was considered as a therapeutic success. An improvement between 20% and 40% was achieved in about 11% of all patients. A subjective improvement between 40% and 60% was observed in 23% percent of the patients, whereas an improvement between 60% and 80% was reported by 18% of the patients. Notably even 18% of all patients showed an improvement from 80% up to 100% equaling a total remission of their symptoms. Only a very small percentage of 2% of the patients reported a worsening of disease burden following radiotherapy.

Next, the effects of different confounding factors, such as age, gender, the number of radiation series, the type and number of fingers affected, as well as the single dose were examined for their influence on the therapeutic success. Therefore, univariate and multivariate analyses were applied. For the analysis, therapeutic success was defined as an improvement of at least 20% as a cut-off. Here, the best response, independent of the time point of data collection (directly after LDRT, as well as 3- and 6-months post LDRT), was applied for analysis. Regarding the potential impact factor age, no correlation between the age and the therapeutic response was found. Moreover, the gender also did not show any correlation to treatment success either. Regarding the number of affected fingers, no correlation between the number of affected fingers and the outcome could be proven. Additionally, for the number of radiation series, there was no significant correlation to the treatment success. The results of the uni- and multivariate analysis are summarized in Table 1.

Nonetheless, there was a positive correlation between the pattern of affection of the fingers with the therapeutic outcome. These findings were true for the univariate regression, whereas no significant results were obtained from the multivariate regression, comparing all parameters. The fingers were separated into thumb and fingers II–V. Here, the affection of solely the thumb versus a mixed affection pattern of the fingers (thumb and fingers II–V) was analyzed for the therapeutic response. Patients with an affection of only the thumb showed a significantly worse outcome compared to patients with a mixed affection of the fingers (*p* = 0.016). Patients with an affection of fingers II–V (without the thumb) showed a significantly better outcome than patients with a mixed affection pattern with the thumb included (*p* = 0.029).

**Figure 2:**
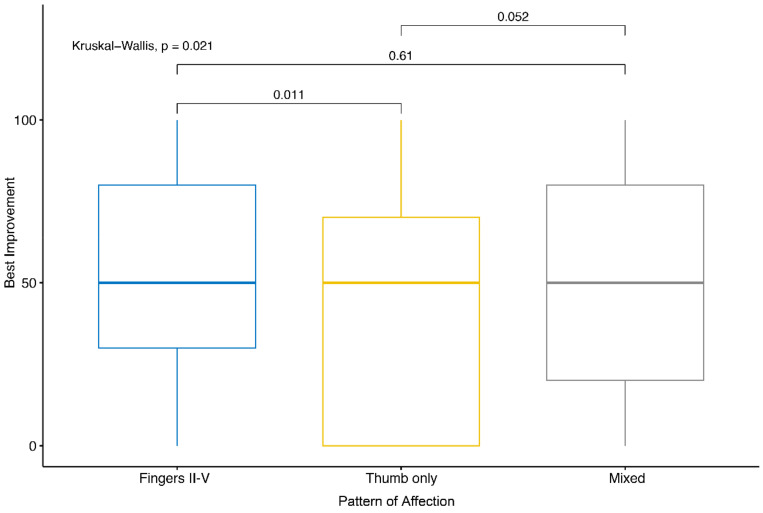
Therapeutic success in dependence of the pattern of affection of the fingers. The Kruskal–Wallis Test was applied to compare and the best improvement of patients in dependence of the affection pattern of the fingers. The patients were grouped according to the affection pattern of the fingers: thumb only, fingers II–V without the thumb, or a mixed affection pattern. Here, the best percentage of improvement achieved was applied for the test independent of the time point of data collection.

For that reason, the impact of the pattern of affection of the fingers on the therapeutic response was analyzed in more detail. Figure 2 summarizes the influence of the affection pattern on the therapeutic success of LDRT.

Patients with an affection of the thumb solely, rate their percentage of improvement in summary significantly lower, than patients with an affection of the fingers II–V without the thumb (*p* = 0.011). Although not statistically significant (*p* = 0.052), an affection of the thumb alone also leads to a worse rating of the pain levels than a mixed affection pattern of the fingers (thumb and fingers II–V). On the contrary, a mixed affection pattern of the fingers does not lead to a worse therapeutic outcome than an affection of only the fingers II–V. In summary, LDRT for osteoarthritis and chronic degenerative joint diseases of the thumb seems to be not as effective as for the other locations of the hand. Nonetheless, the best response rates still reached a total remission in some cases with an affection of the thumb.

**Figure 3:**
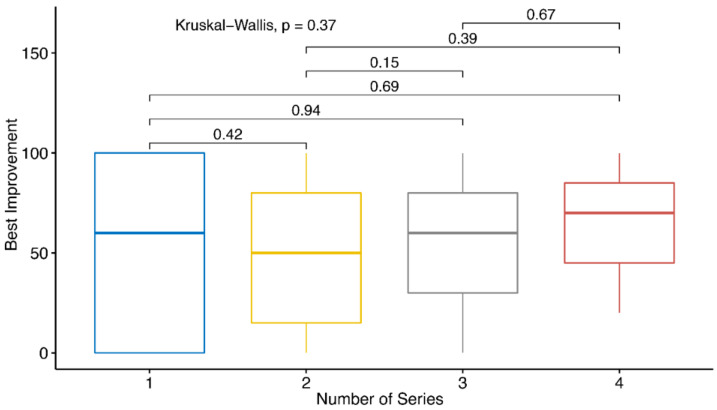
Therapeutic success in dependence of the number of series of LDRT applied. The Kruskal–Wallis Test was applied to compare the best improvement of patients in dependence of the number of radiation series (1, 2, 3 or 4) received. Here, the best percentage of improvement achieved was applied for the test independent of the time point of data collection.

As many patients demand more than one series of LDRT, the influence of the number of series on the therapeutic outcome was analyzed in more detail, as a reduction in the series would be desirable in the context of radiation protection. Therefore, the patients were grouped depending on the LDRT series received and their therapeutic responses were compared. These results are summarized in Figure 3.

Patients receiving only one series of LDRT report a median improvement of about 52% in comparison to the pre-therapeutic pain level. Nonetheless, the reported best improvement of the patients receiving only one series shows a broader spread (between 0% and 100%) than patients receiving more series of LDRT. When getting two or three series of LDRT, the patients report a median best improvement of about 48% and 55%, respectively. An even stronger pain reduction was achieved in patients receiving four series of LDRT with a median improvement of about 63%. In summary, these data indicate that more series of LDRT induce stronger pain-relieving effects in the patients. Nonetheless, the differences in the levels of improvement are weak (*p* = 0.37).

**Figure 4:**
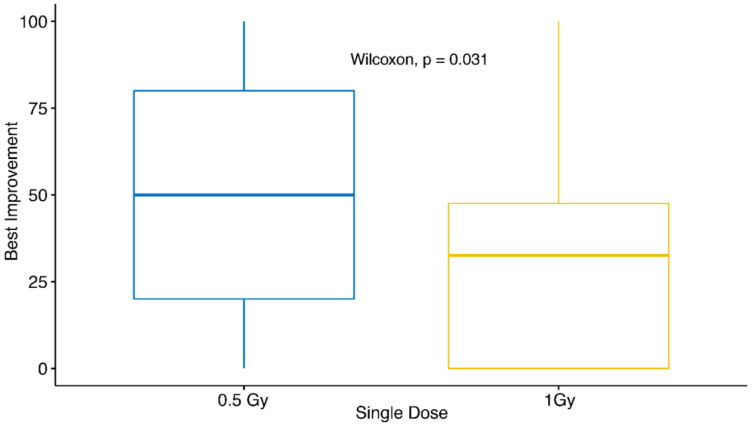
Therapeutic success in dependence of the applied single dose of LDRT. The Wilcoxon Test was applied to compare the best improvement of patients in dependence of the single dose per fraction (0.5 Gy or 1.0 Gy) of LDRT. Here, the best percentage of improvement achieved was applied for the test independent of the time point of data collection.

Despite the fact that LDRT is commonly applied for the therapy of chronic degenerative and inflammatory diseases, the German guidelines still do not suggest uniform recommendations for the best single dose, still suggesting doses ranging between 0.5 Gy and 1.0 Gy [23]. Therefore, we analyzed the effect of the single dose on the therapeutic outcome in more detail (Figure 4).

Even though a significant difference in the therapeutic outcome between patients treated with 0.5 Gy and 1.0 Gy was not observable in the uni- and multivariate analyses presented before, we performed an additional direct comparison for the two different single doses. Here, the best therapeutic response independent of the time point of data collection was applied for the analysis. Patients with a single dose of 0.5 Gy have a significantly better therapeutic outcome (*p* = 0.031) (median improvement: about 50%) than patients irradiated with a single dose of 1.0 Gy (median improvement: about 30%). This finding strongly indicates that a dose of 0.5 Gy is sufficient for a satisfying therapeutic outcome and higher single doses are not required.

**Figure 5:**
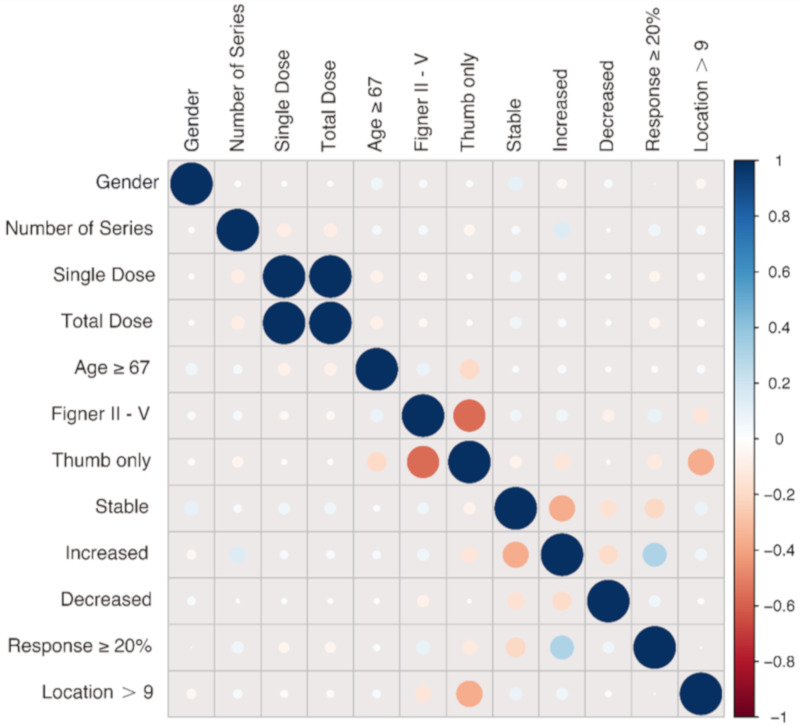
Correlation plot summarizing the analyzed impact factors. The size of the dots states the strength of the correlation while the color reveals a positive (blue) or negative (red) correlation between two factors. The plot visualizes the correlation of all investigated impact factors on treatment success.

Finally, we examined the impact of confounding factors for the relationship of the single dose and the best improvement by logistic regression. These factors include: the gender, the number of radiation series, the total dose, the age, an affection of the finger II–V or solely the thumb. In addition, we analyzed if the pain level of the patients was stable, increasing or decreasing over the follow-up time of six months, if the patients had a therapeutic response better than the cut-off of 20% of improvement and if the number of affected joints influenced the outcome. Figure 5 summarizes and visualizes these correlations.

There is a weak positive correlation between the gender male and a stable pain level during the follow-up period (r = 0.1). This means that men tend to have a more stable therapeutic outcome. The number of the series has a positive correlation to an increased pain level over the time of the follow-up period (r = 0.14). So, patients with an increasing pain level over the follow-up time demand more radiation series. Additionally, patients with a response rate better than 20% demand more radiation series, as there is also a weak positive correlation between the number of the series and the response rate better than 20% (r = 0.07). Moreover, an age over 67 years correlates negatively to an affection of the thumb (r = −0.2). This indicates that younger patients are more likely to develop osteoarthritis, mainly of the thumb. On the other hand, there is a weak positive correlation between the age and an affection of the fingers II to V (r = 0.09), indicating that older patients have a higher risk of developing osteoarthritis/chronic degenerative joint disease of the fingers without an affection of the thumb. This affection pattern of the fingers is also positively correlated to a positive therapeutic response (response ≥ 20%) (r = 0.1), further supporting our data that an affection of the fingers II to V leads to a better therapeutic outcome (Figure 2). In addition, an affection of the fingers II to V is negatively correlated with an affection of more than nine independent locations (location > 9) (r = −0.14). This negative correlation is even stronger when the locations are correlated to the affection of the thumb (r = −0.37), suggesting that an affection of the thumb is more likely to be independent of the rest of the fingers. Moreover, the affection of the thumb correlates negatively to an increasing pain level over the follow-up time (r = −0.13), as well as to a positive therapeutic response (response ≥ 20%) (r = −0.11). Therefore, patients with an affection of the thumb are more likely to not respond to LDRT, but if they are responding, their pain level seems not to increase over the period of six months post LDRT. Interestingly, an affection of more than nine locations correlates positively with a stable response over the follow-up time (*p* = 0.08). A stable response over the follow-up period also correlates negatively to a therapeutic response better than 20% of improvement (r = −0.2). An increased pain level over the follow-up period on the other hand shows a strong positive correlation to a therapeutic response over 20% (r = 0.3).

## 3. Discussion

LDRT is well established for both orthovoltage as well as megavoltage radiotherapy techniques. To our knowledge, this is the biggest study selectively investigating the effect of low dose radiotherapy using orthovoltage therapy in chronic degenerative joint disease of the fingers. LDRT has been applied as an alternative treatment option for patients suffering from painful arthritis for several decades. It is a reliable tool in the treatment of patients with osteoarthritis and other degenerative joint diseases due to a vast body of data attesting its efficacy [24,25,26,27], even though the first randomized trials show less promising results [21,22]. However in these trials, clinical data provided on irradiation volumes, at least with regard to the treatment of arthrosis of the thumb, raise concerns about their suitability [28]. Furthermore, both studies have several limitations, such as the short-term follow-up of only 3 months, low patient numbers, and a too optimistic prognosis assessment for the success evaluation [29]. On the other hand, a recently published prospective trial demonstrated that LDRT is leading to a significant pain relief and gain of functionality in patients suffering from refractory finger osteoarthritis [15]. The data obtained from our presented analysis of 483 patients confirm the positive effect of LDRT on degenerative joint disease and osteoarthritis of the fingers with the majority of patients showing an improvement directly after therapy that even lasts several months. Moreover, only 2% of the patients reported a worsening of the pain and 27% a pain relief less than 20% following RT, indicating a rather small proportion of non-responders. Regarding the fact that LDRT is commonly applied for patients that are refractory for classical treatment strategies, this finding is even more impressive. Nonetheless, in the presented study, the effectiveness of LDRT is only confirmed by the subjective improvement of the pain levels of the patients, which is due to the retrospective character of this study. Even though the presented results are promising, future studies should focus on objective and clinically validated methods, such as functionality tests and questionnaires.

Our results did further show a tendency towards a better treatment result following LDRT for patients undergoing a higher number of radiation series. Considering the fact that patients were undergoing radiotherapy for a third or fourth series only on an individual basis and on individual request, this fact supports our findings, as only patients who profited in the past would demand for further treatment. One disenchanting factor that needs to be discussed is the fact that the worsening of therapeutic response can be observed even after the initial promising treatment response. This possible scenario should be discussed with the patient prior to radiation. In addition, the further improvement of the pain levels that can be achieved by applying several radiation series is only small, as the difference in the level of improvement is about 10% when comparing patients receiving one series with patients receiving four series. Therefore, the benefit of additional LDRT series should be balanced to the potential radiation risk [30].

Further subgroup analyses showed no significant difference in the response for the number of affected fingers. Patients suffering from multiple painful locations still can expect a positive treatment effect. Neither an advanced age, nor the gender showed a significant impact on treatment outcome. Regarding the age factor, the correlation plot (Figure 3) indicates a positive correlation with an involvement of fingers II–V and a negative correlation between the age and the involvement of the thumb. This indicates that the involvement of the thumb is more relevant at a younger age, possibly rather by destructive than inflammatory processes resulting from an overuse of the thumb. On the other hand, the positive correlation between the rising age and the involvement of the fingers II–V indicates that the prevalence for systemic inflammatory processes, such as in arthritis, is rising with the age [31]. The here presented results show that patients with involvement of the thumb have a worse therapeutic outcome. Perhaps the rather destructive phenotype in patients with an affection of the thumb is less accessible for the anti-inflammatory effects of LDRT. Moreover, the thumb is more excessively strained than the other fingers, possibly resulting in a state less accessible for a therapeutic intervention by LDRT. Additionally, the analgesic effects of LDRT could last for a shorter period of time due to the lack of conscious or unconscious immobilization. A combined usage of orthoses, limited in time, and LDRT might be an option to increase therapeutic success [32].

In our cohort of patients, receiving a single dose of 1.0 Gy, did show a significantly worse outcomes when compared to those receiving a single dose per fraction of 0.5 Gy, strengthening the findings that a LDRT series with a single dose per fraction of 0.5 Gy is at least as effective as with 1.0 Gy [33]. Consistent with this finding, preclinical studies suggest that a single dose of 0.5 Gy induces stronger anti-inflammatory effects than a single dose of 1.0 Gy [20,34]. These anti-inflammatory effects could, at least in part, be responsible for the pain relieving effects of LDRT. For this reason, more than 95% of the patients in this study received LDRT with a single dose of only 0.5 Gy. In summary, our results strongly indicate the application of lower single doses, according to the ALARA principle of radiation protection [35].

Even though our study presents promising results, a possible placebo effect of LDRT cannot be ruled out. Nonetheless, a placebo effect seems to be unlikely, considering the high percentage of patients who have been proven refractory to multiple preceding treatments and the high number of patients reporting a pain reduction. In our analysis, we chose to present the best improvement achieved for each patient during follow up to be capable of showing clear tendencies regarding an overall treatment success. Data describing individual long-time development can only be acquired applying prospective studies.

Irrespective of the retrospective setting of the study, a good overall response rate following the application of LDRT has been reported for patients suffering from osteoarthritis and degenerative joint disease of the fingers. In summary, this treatment modality delivers satisfying results independent of patient’s age or the number of localizations. Patients with an affection of the thumb can be expected to show worse results. Anyhow, satisfying improvement rates were still achieved in these patients. In addition, one series of LDRT with a single dose of 0.5 Gy resulted in better pain-relieving effects than higher radiation exposure did.

In the future, randomized trials are urgently needed, fulfilling high standards to increase the evidence level, such as high patient numbers, follow-up times of up to one year, a defined patient cohort and an evaluation of patient outcome not only by subjective pain reduction but also by objective immunological and rheumatological parameters, as well as functionality tests and questionnaires [29].

## 4. Materials and Methods

### 4.1. Patient Cohort

Between November 2004 and February 2019, 483 (98 men, 385 women) patients with degenerative and inflammatory joint disease of the fingers, including the thumb, received LDRT at the Department of Radiation Oncology of the Universitätsklinikum Erlangen. The patients were referred by their treating orthopedic specialists. All patients have received several therapeutic options before undergoing LDRT, as an alternative treatment modality. The majority of the patients did not receive full clinical, rheumatologic diagnostics before therapy. In general, the patients have been assigned to our clinic with a chronic degenerative joint diseases of the fingers, displaying primarily symptoms of hand osteoarthritis. At the time of radiotherapy, the median age of the patients was 67 years with a median age of 64 years for the women and 67 years for the men. In summary, 461 patients underwent radiotherapy with a single dose of 0.5 Gy (total dose of 3 Gy), whereas 22 patients underwent radiotherapy with a single dose of 1.0 Gy (total dose of 6 Gy). As preclinical studies indicate stronger anti-inflammatory effects with a single dose of 0.5 Gy, most patients underwent RT with 0.5 Gy. Furthermore, 29 patients received one series of LDRT, 410 patients received two series and 41 patients underwent three series of LDRT. Only three patients underwent four consecutive series of LDRT. Table 2 provides a summary of these patient characteristics.

Written informed consent was obtained from all patients before LDRT. In addition, patients provided informed consent for the retrospective analysis of their data, as well as the publication of the data in a pseudonymized manner.

### 4.2. Treatment

All patients were undergoing radiotherapy applied with an orthovoltage technique. Radiotherapy was applied using a Stabilipan machine (180 kv, 20 mA, 0.2 mm Cu filter, focus skin distance 40 cm) until September 2016. Afterwards an X-strahl machine was used (180 kV, 10 mA, 0.2 mm Cu filter, focus skin distance 50 cm). No qualitative differences can be expected concerning radiation quality. The single fields applied for both machines ranged from 6 × 8 cm to 10 × 8 cm and 10 × 15 cm, according to the field needed for different numbers of treated fingers. The fields were positioned directly on the affected fingers with a determination of the dosage at the middle of the joint. One radiotherapy series consisted of six single fractions (single dose: 0.5 Gy or 1.0 Gy) delivered over three weeks with an interfractional radiation-free interval of at least two days (total dose: 3 Gy or 6 Gy). Patients not showing an improvement of the pain or not being subjectively satisfied six weeks after the end of the first series underwent a consecutive second series 12 weeks following the first. The application of further series occurred only on an individual basis.

### 4.3. Measurement of Therapeutic Outcome

The data on patient’s therapeutic outcome was collected prospectively exclusively for clinical routine and afterwards analyzed retrospectively. The retrospective use of the patient’s data is covered by an allowance by the Ethics Committee of the Friedrich-Alexander-Universitäts Erlangen-Nürnberg (Ref. 238_ 20 Bc, FingerRetroRad trial). The study was performed in accordance with the 1964 Declaration of Helsinki and its later amendments. The outcome in the present study was measured by quantifying the subjective pain reduction. Therefore, patients rated their subjective pain reduction in terms of percentage of improvement with reference to their initial pain status before LDRT. The interrogation of patients was performed directly after the last radiotherapy session and during the follow-up appointments 8 to 12 weeks later, as well as six months after LDRT. For all patients, the number of affected fingers was investigated. Further investigation of separate single joints of one finger were not considered. Patients were differentiated into separate groups, depending on whether they showed an affection of only the thumb or an involvement of the fingers II–V or both. Analyses were performed with the subjective ratings obtained at the last session of RT, three months post RT and six months post RT, if available.

### 4.4. Statistical Analysis

The Kruskal–Wallis (α = 0.05) tests were used to evaluate the difference among groups on the best improvement during therapy using *ggpubr* (version 0.2.5) (*p* < 0.05). Univariate and multivariate logistic regression was used to evaluate the adjusted association of the single dose and the best improvement (thumb only, fingers II–V, both) (*glm.fit function*). A correlation analysis of the factors in each group was performed by *ggcorrplot* (version 0.1.3). Data management was performed using the IBM SPSS software for MS Windows (SPSS Inc. Chicago, IL, USA, version 21). All analyses were carried out using R version 3.6.1 (R Foundation for Statistical Computing) and related packages. *p* ≤ 0.05 was considered to be statistically significant.

## Figures and Tables

**Table 1:** Univariate and multivariate logistic regression analyzing the clinical factors influencing the treatment success following LDRT.

	Univariate Regression			Multivariate Regression		
Parameter	OR	95% CI	*p*-Value	OR	95% CI	*p*-Value
Gender (male vs. female)	1.00	0.621–1.645	0.997	0.998	0.614–1.652	0.993
Number of Series	1.473	0.905–2.45	0.126	1.403	0.856–2.354	0.188
Single dose (1 vs. 0.5 Gy)	1.689	0.682–4.010	0.239	1.642	0.650–3.985	0.277
**Finger II–V only vs. mixed**	1.720	1.070–2.841	**0.029**	1.208	0.621–2.341	0.576
**Thumb only vs. mixed**	0.616	0.414–0.912	**0.016**	0.627	0.340–1.125	0.125
Number of fingers (>9 vs. ≤9)	0.996	0.568–1.806	0.988	0.764	0.374–1.578	0.462
Age (≥67 y vs. <67 y)	1.10	0.742–1.635	0.636	0.996	0.641–1.460	0.871

**Table 2:** Patient characteristics.

Factor	Category	Total (*n* = 483)	
		*n*	(%)
Age	≥67	269	55.69
	<67	214	44.31
Gender	Male	98	20.29
	Female	385	79.71
Pattern of involvement	Finger II–V	120	24.84
	Thumb	236	48.86
	Both	127	26.3
Single dose	0.5 Gy	461	95.45
	1 Gy	22	4.55
Total dose	3 Gy	461	95.45
	6 Gy	22	4.55
Number of Series	1	29	6
	2	410	84.89
	3	41	8.49
	4	3	0.62

## Abbreviations

LDRT	Low-dose radiation therapy
DMARDs	Disease-modifying anti-rheumatic drugs
NSAIDs	Non-steroidal anti-inflammatory drugs
Gy	Gray
